# Near-infrared spectroscopy combined with pattern recognition algorithms to quickly classify raisins

**DOI:** 10.1038/s41598-022-12001-1

**Published:** 2022-05-13

**Authors:** Jiawei Guo, Cheng Chen, Chen Chen, Enguang Zuo, Bingyu Dong, Xiaoyi Lv, Wenzhong Yang

**Affiliations:** 1 College of Software, Xinjiang University, Urumqi, 830046 China; 2 College of Information Science and Engineering, Xinjiang University, Urumqi, 830046 China; 3 Xinjiang Aiqiside Testing Technology Co, Urumqi, 830011 China; 4 Xinjiang Cloud Computing Application Laboratory, Karamay, 834099 China

**Keywords:** Chemical engineering, Applied optics

## Abstract

With the development of commodity economy, the emergence of fake and shoddy raisin has seriously harmed the interests of consumers and enterprises. To deal with this problem, a classification method combining near-infrared spectroscopy and pattern recognition algorithms were proposed for adulterated raisins. In this study, the experiment was performed by three kinds of raisins in Xinjiang (Hongxiangfei, Manaiti, Munage). After collecting and normalizing the spectral data, we compared the spectra of three kinds of raisins. Next the principal component analysis (PCA) was preformed to compress the dimension of the spectral data, and then classification models including support vector machine (SVM), multiscale fusion convolutional neural network (MCNN) and improved AlexNet were established to identify raisins. The accuracy of SVM, MCNN, and improved AlexNet is 100%, 92.83%, and 97.78% respectively. This study proves that near-infrared spectroscopy combined with pattern recognition is feasible for the raisin inspection.

## Introduction

Raisin is a kind of nutritious and diverse agricultural product, which is rich in nutrients such as sodium, iron, calcium, and dietary fiber^1^. Studies have found that eating raisins three times a day can significantly reduce blood sugar levels, systolic and diastolic blood pressure, thereby reducing the risk of diabetes and cardiovascular and cerebrovascular diseases in consumers^2^. Furthermore, compared with carbohydrate foods with the same calorie, raisins can effectively reduce cholesterol levels and have anti-inflammatory and anti-cancer effects. Therefore raisin is beneficial for promoting body and heart health and preventing many chronic diseases^3^. At present, raisin is widely used in food processing such as making various snacks or adding staple foods^4–6^. Due to the differences in the variety, origin, and drying process, the taste, nutritional content, and commercial value of raisins are very different^7–9^. In addition, there is a serious problem of fake raisin varieties and inferior quality in the market at present, which have a bad impact on the healthy development of the raisins market^10^

At present, the method for the classification of raisins mainly relies on the extraction of texture features from raisins' images. For example, Khojastehnazhand et al. used the gray horizontal run length matrix (GLRM) combined with SVM to classify 15 kinds of raisins and got 69.78% accuracy respectively^11^. Navab·Karimia et al. used a method based on machine vision combined with ANN and SVM to identify golden bleached raisins among a variety of raisins, and the final accuracy rate of golden bleached raisins was 92.71%^12^. However, the raisin' size is small and has fewer image features that can be extracted. At the same time, the photographing process is susceptible to inevitable factors such as lighting, equipment, and pixels. Therefore, this method has some limitations that restrict its wide application. To overcome the limitations of the above method, this research proposes a classification method based on near-infrared spectroscopy.

Since mature grape will form a waxy layer on the surface during the process of dehydration, the waxy layer formed by different varieties of grapes under a variety of dehydration methods will exist differences in lipids, polyphenols, and trace elements^13^. Therefore, raisin skin is selected as the object of study.

Near-infrared spectroscopy, as a commonly used material quantitative analysis and chemical structure detection tool, has the characteristics of low energy and very high efficiency. In addition, it also has the advantages of unlimited sample form, less dosage, and no damage to the sample^14^. At present, near-infrared spectroscopy has a wide range of applications in various research fields, such as medical diseases, food detection, and gem identification^15–18^.

Machine learning is a data analysis technique. It selects appropriate algorithms through data, automatically summarizes logic and rules, and make predictions based on the generalized model^19^. Deep learning algorithm which is a type of machine learning algorithm, is generally composed of one or several layers of deep neural networks^20^. Deep learning algorithm has been applied to research in many fields^21^. This research optimized the deep learning algorithm and made the model more suitable for the classification of near-infrared spectroscopy data. To improve performance, Batch normalization (BN) was added to the model in this experiment. BN is a method for optimizing neural networks. It can speed up the convergence speed of model training, make the training process more stable, and avoid gradient explosion or gradient disappearance. It also has the function of regularization^22^.

In this study, we used near-infrared spectroscopy combined with pattern recognition algorithms to classify three kinds of raisins: Hongxiangfei, Manaiti and Munageto. First, we collected the spectral data from the raisin pericarps and then used PCA to extract the characteristics of the spectral data of the pericarps. Finally, we constructed SVM, MCNN and improved AlexNet model for classification.

## Experimental materials and methods

### Sample preparations

Three kinds of raisins: HongxiangFei, Manaiti and Munage were selected in the experiment. Among them, Hongxiangfei and Munage raisins were purchased from Shangyao dry and fresh fruit specialty boutiques in Urumqi, Xinjiang, China. Manaiti raisins were purchased from Urumqi Xiyu Baza E-Commerce Co. Ltd. The origin of Hongxiangfei is Hami, and the origin of Manaiti and Munage are Turpan. The pericarps were separated from raisin. Then, the skin samples were placed in a YG747 fast constant temperature oven (Changzhou First Textile Equipment Co., Ltd.) for two hours and the temperature was set to 100 °C. Afterwards, they were packaged in ziplock bags.

### Near-infrared spectroscopy measurement

The experimental measurement used a VERTEX 70 FT-IR spectrometer from Germany with a resolution of 8 cm^−1^; the scanning range was 4000–11,000 cm^−1^; OPUS 65 software was used to measure the atmospheric background data before each measurement, which scanned on zinc slenide; the atmospheric compensation parameter was CO_2_ compensation and the number of scans was 16 times. To reduce the influence caused by random errors such as noise of spectrometer and the difference of environmental humidity, the measurement was repeated four times for each sample and the average value was taken. In addition, to reduce the influence of electronic drift and other factors, the near-infrared spectroscopy used Rubberband baseline correction, and the number of baseline points was set to 64. In the end, Hongxiangfei obtained 59 average spectra, and Marquise and Munage each obtained 60 average spectra.

### Method introduction

PCA is a data mining technique in multivariate statistics. It selects a small number of new variables to replace the original old variables without losing the main spectral information. It not only solves the difficulty of being unable to analyze due to overlapping bands but also helps in the interpretation, understanding, discrimination and clustering of measurement data^23^. In this study, PCA was used to reduce the dimensionality of the spectral data.

SVM is a supervised binary generalized linear classifier. The data are classified by constructing the best hyperplane. SVM is a learning algorithm for small samples. Its essence is to mine the classification information hidden in the data to the maximum in the limited samples. In addition, the non-linear problem in the original space is transformed into the linear problem in the high-dimensional space through the non-linear transformation. It not only guarantees good promotion ability, but also does not increase the algorithm complexity. SVM has been widely used in food research^24^. In summary, we chose SVM as the first algorithm of multivariate classification.

Convolutional Neural Network (CNN) is a deep learning structure for feature extraction, classification, and regression^25^. In the literature, CNN has been widely applied to food^26^. Combining the characteristics of spectral data, this study designed and evaluated a CNN structure for classification.

The AlexNet model is a deep convolutional neural network proposed by Alex Krizhevsky and others at the University of Toronto. AlexNet has more parameters and convolutional layers so that it is more efficient to extract features. At the same time, AlexNet uses the ReLU activation function and Dropout to reduce the risk of overfitting, which not only greatly improves the performance of the model but also improves the recognition accuracy^27^. We made some adjustments to AlexNet to better adapt to the spectral data^28^.

### Method evaluation indexes

In order to evaluate the classification effect of the model accurately and comprehensively, we used three common model evaluation indexes, namely accuracy, sensitivity and recall rate.

Accuracy represents the percentage of the total sample that is predicted correctly, and the formula is as Eq. (1)

1 $$Accuracy = \frac{TP + TN}{{TP + FP + FN + TN}}$$

Accuracy is the simplest and most intuitive evaluation index in the classification problem, but it has obvious defects. When the proportion of different types of samples is very uneven, the larger samples have a greater impact on the accuracy. Therefore, it is not sufficient only through the accuracy to evaluate the model.

Precision represents the proportion of the number of correct pictures to the total number of positive predictions. The formula is as Eq. (2)

2 $$Precision = \frac{TP}{{TP + FP}}$$

Recall represents the probability that a correct sample is predicted to be positive in all the correct numbers, and the formula is as Eq. (3)

3 $$Recall = \frac{TP}{{TP + FN}}$$

## Result and analysis

### Spectral analysis

**Figure 1:**
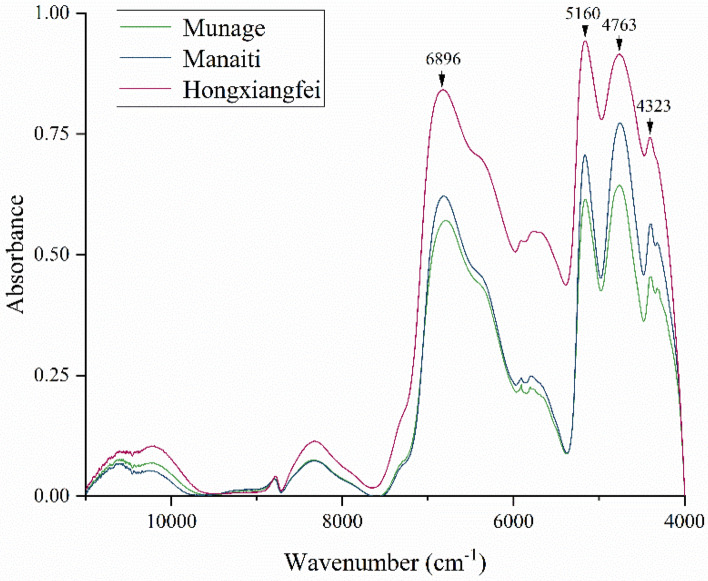
Average spectroscopy of raisin pericarps.

We normalized the spectral data of raisins to the [0,1] range. The resulting spectra are shown in Fig. 1. Three spectral lines represent the average near-infrared spectra of Hongxiangfei raisins, Manaiti raisins, and Munage raisins in the range of 4000 cm^−1^ to 11,000 cm^−1^, respectively. As shown in the figure, the spectra are similar but different in intensity. Hongxiangfei raisins have the highest normalized spectral intensity, Munage raisins have the lowest normalized spectral intensity, and the spectral intensity of Manaiti raisins lies between the two. According to relevant literature, different infrared absorption bands and corresponding substances are indicated in Table 1^26,29–31^.

**Table 1:** Chemical bond information corresponding to spectral characteristic peaks of raisin pericarps^26,29–31^.

Wavenumber (cm^−1^)	Chemical Bond information
4323	C–H bending of the lipids
4763	C–O stretching and O–H deformation
5160	C=O groups of the carbohydrates
6896	O–H of the flavonoids

Spectral characteristic peaks of the three groups of samples are mainly distributed at 4323, 4763, 5160, 6896 and so on. Combined with the peak material distribution analysis, the variety types of raisins and the drying treatment methods cause the difference in lipid content in the waxy layer on the surface of the raisins^32^. In addition, the processing method of raisins is also the main reason for the difference in the content of brass and phenolic acid in raisins^33^.

### Data processing results

#### PCA dimensionality reduction results

**Figure 2:**
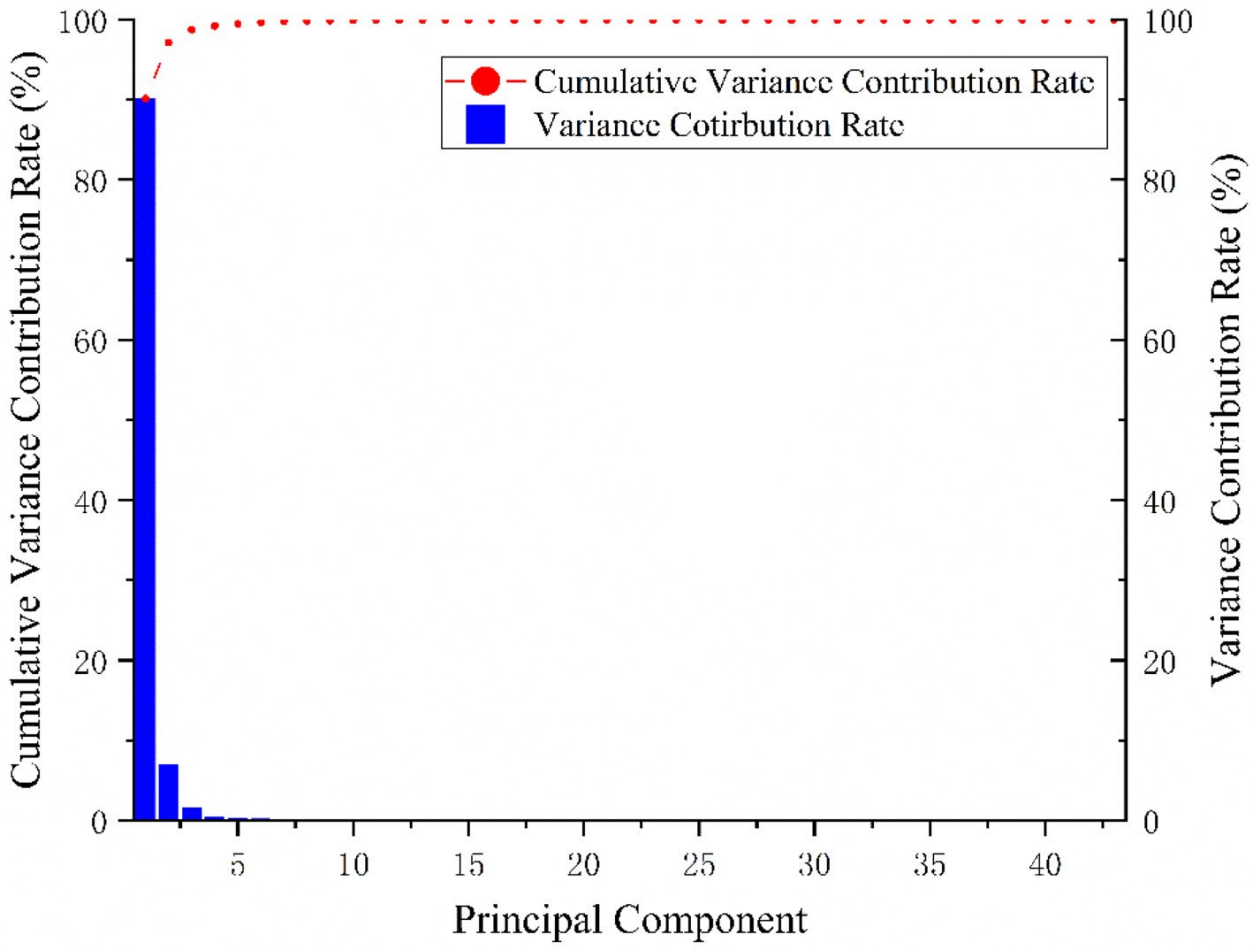
Variance contribution of the principal component.

The variance contribution rate after PCA dimensionality reduction is shown in Fig. 2. The cumulative variance contribution rate of the first 25 principal components exceeded 99.98%^34^. The above results show that the 25-dimensional feature variables obtained by PCA can basically explain all the information of the original variables. So this study used the first 25 features for subsequent analysis^23^. After feature extraction, the data were randomly divided into training set, test set and validation set at a ratio of 6:2:2. We used SVM, MCNN and improved AlexNet for model training. To ensure the stability of the experimental results, each model was run five times. The final result was the average of five runs.

#### SVM

**Figure 3:**
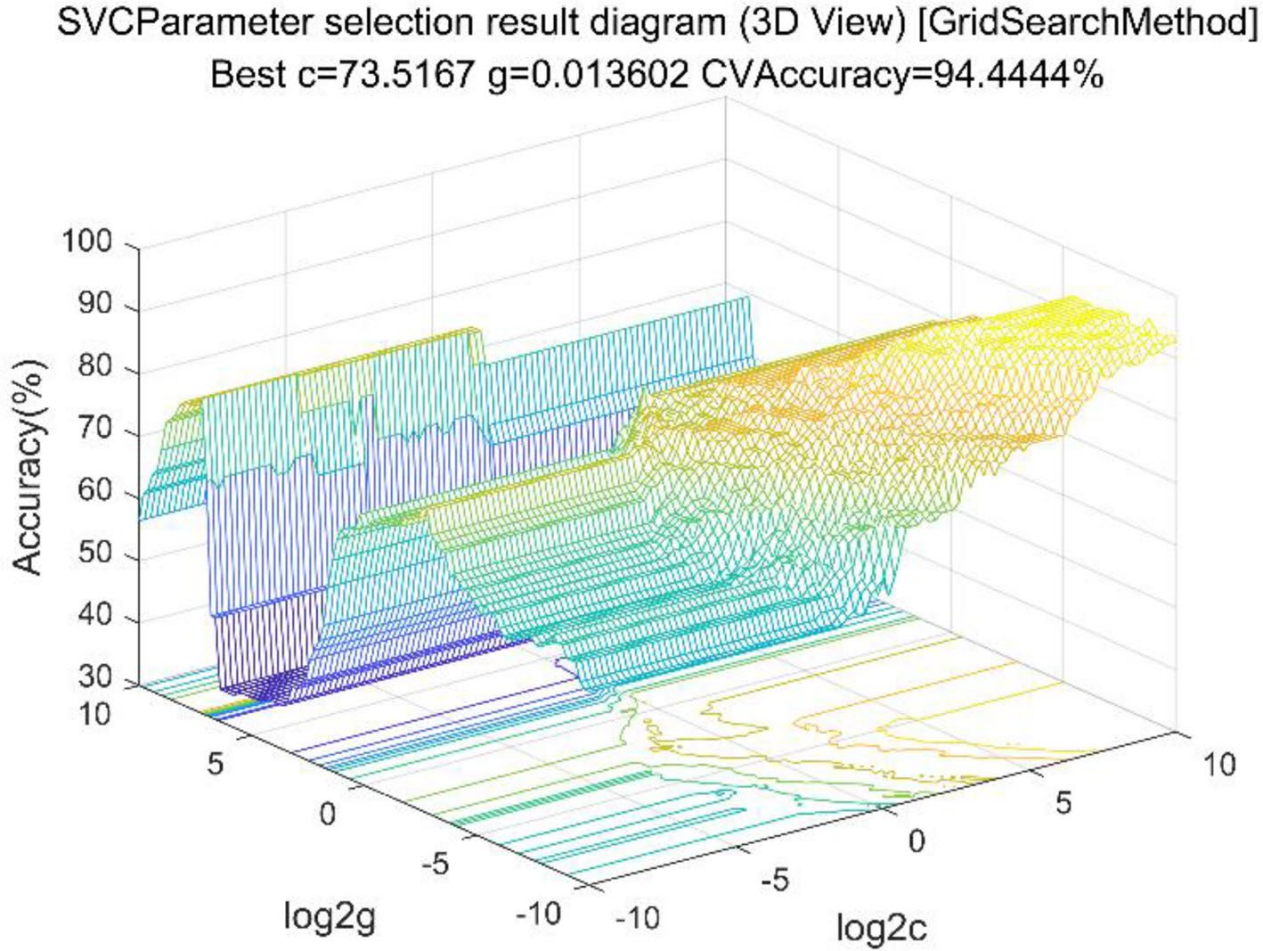
SVC Parameter selection result.

We selected the radial basis function as the kernel function. By conducting a grid search, optimal weight facstors were determined. Grid optimization is an exhaustive search method. It loop through all the values in the range of parameters c and g and compare their accuracy to determine the best c and g. In this study, the ranges of the parameters c and g were [2–10]^35^. When the feature dimension is 25 dimensions, the selection results of parameters and their accuracy are shown in Fig. 3.

The best c was 0.75786 and g was 0.25. In this study, 5, 10, 15, 20, 25, 30, 35, 40 characteristics were selected to classify the raisins.

#### MCNN

**Figure 4:**
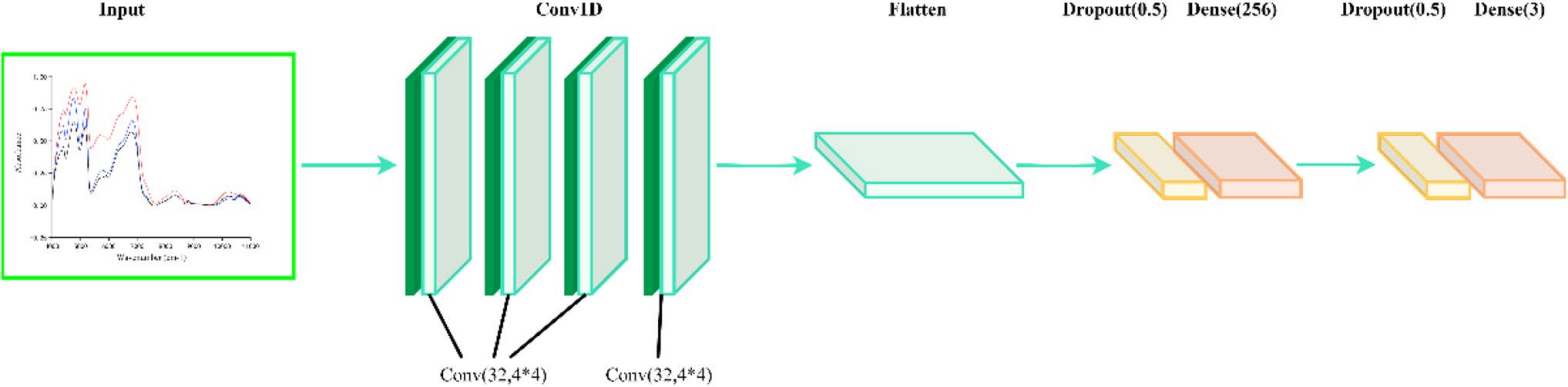
The MCNN model.

The structure of MCNN is shown in Fig. 4. There were six hidden layers of MCNN in this experiment: three convolutional layers, a flatten layer and two fully connected layers. In order to prevent over-fitting and speed up the convergence speed, a BN layer was added before each convolutional layer^36^. The number and size of convolution cores in the convolution layer and the parameters of other layers are shown in Fig. 4. At the same time, two dropout layers were inserted before the two fully connected layers and the corresponding dropout rates were set to 0.5; LeakyReLU was selected as the activation function; alpha was 0.1; Adam was the optimizer; the learning rate was set to 1 × 10^–5^ and the training batch size was set as 64; the number of training times was set for 200 times.

#### The improved AlexNet

**Figure 5:**
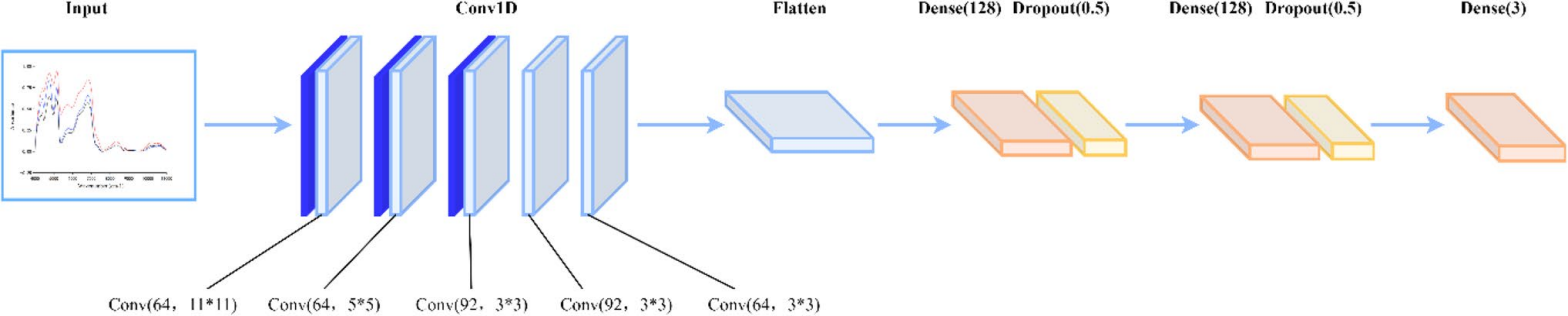
The adjusted AlexNet model.

The improved AlexNet structure is shown in Fig. 5. It had five convolutional layers, a flatten layer, and three fully connected layers. The number and size of convolution cores in the convolution layer and the parameters of other layers are shown in Fig. 5. Three BN layers were added before the first three convolutional layers, and two dropout layers with dropout probabilities of 0.5 were added between the first two of the three fully connected layers; the activation function was ReLU; the learning rate was 1 × 10^–7^ and the batch size for training was set to 32. The training procedure were repeated 200 times. The experimental results are shown in the Table 2.

**Table 2:** Test set experimental results of SVM, AlexNet and MCNN.

Number of PCA components	SVM			AlexNet			MCNN		
	Accuracy (%)	Precision (%)	Recall (%)	Accuracy (%)	Precision (%)	Recall (%)	Accuracy (%)	Precision (%)	Recall (%)
5	94.28	100.00	91.67	80.32	81.87	80.19	80.24	80.05	80.03
10	94.28	85.45	100.00	97.15	97.63	96.71	85.71	86.23	85.85
15	96.11	90.75	91.28	93.55	95.56	90.24	88.75	86.11	84.49
20	100.00	100.00	100.00	100.00	100.00	100.00	94.28	95.23	94.44
25	97.22	100.00	100.00	93.33	94.62	93.28	92.22	92.63	93.50
30	93.33	80.64	96.67	91.42	93.33	91.67	91.42	93.33	91.67
35	94.33	91.28	96.28	90.56	92.41	90.84	90.56	90.73	90.43
40	93.33	81.82	90.15	90.89	94.85	88.76	89.56	90.47	85.56

## Discussion

**Figure 6:**
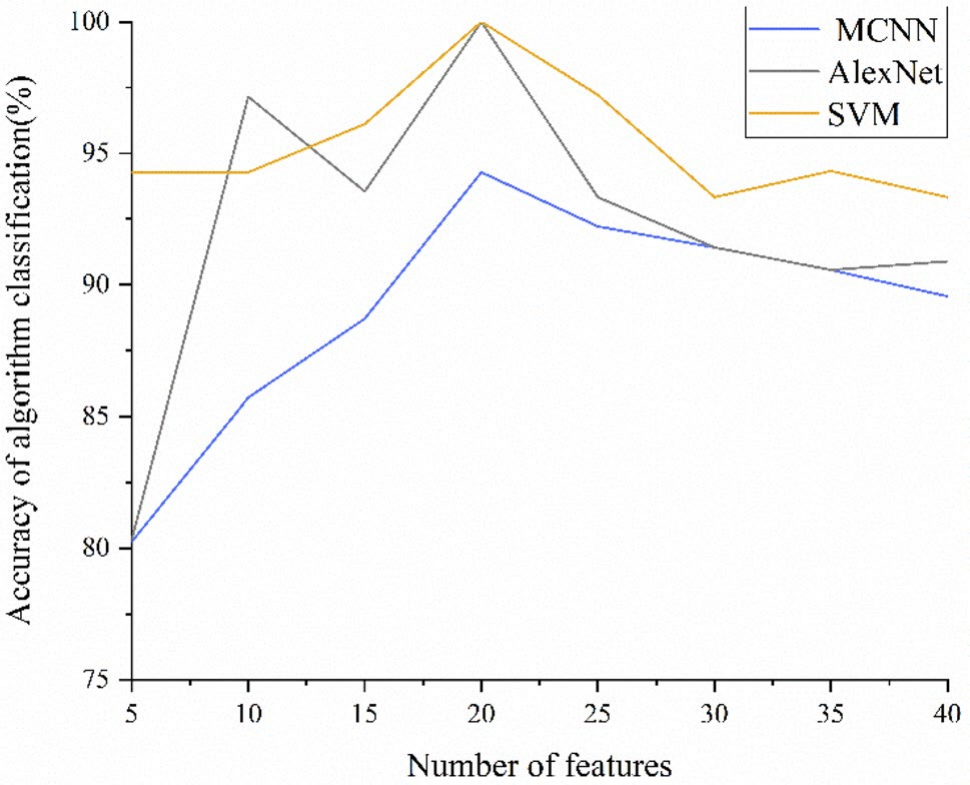
Test set accuracy of SVM, AlexNet and MCNN.

**Figure 7:**
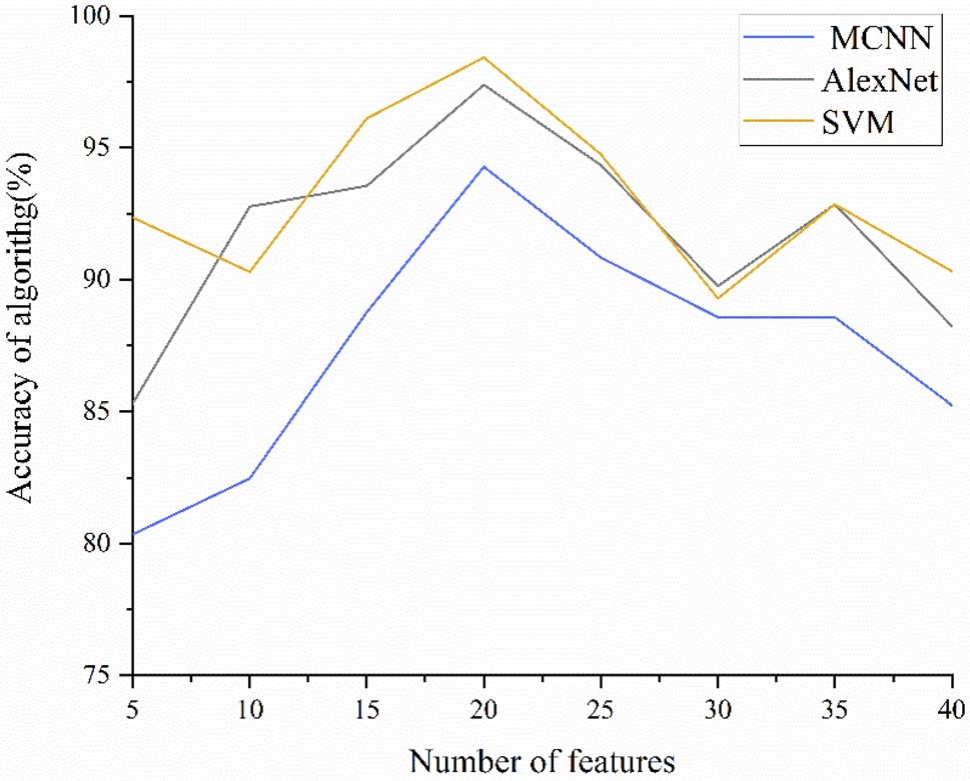
Validation set accuracy of SVM, AlexNet and MCNN.

In this study, we used near-infrared spectroscopy combined with pattern recognition algorithms to quickly and accurately identify three kinds of raisins from different origins. We used SVM, MCNN and improved AlexNet for classification. The accuracy, precision and recall of test set and verification set are shown in Tables 2 and 3. The accuracy of the test set is shown in Fig. 6. The results show that the accuracy of the test set improves with the increase of the number of features when selecting 5, 10, 20, 25 features. When selecting 20 features, the accuracy, precision and recall of the three models are the highest, and SVM, AlexNet get 100% accuracy respectively. After that, with the increase of the number of features, the accuracy decreases slightly, but gradually tends to be stable. The accuracy of the validation set is shown in Fig. 7. The accuracy trend of the verification set is similar to that of the test set. The reason for this trend may be that the number of features is small so that the model is underfitted when selecting 5, 10 features, while when selecting 40 features, some interference information is introduced while the number of features increases, resulting in a little decrease in the accuracy of the verification set.

**Table 3:** Validation set experimental results of SVM, AlexNet and MCNN.

Number of PCA components	SVM			AlexNet			MCNN		
	Accuracy (%)	Precision (%)	Recall (%)	Accuracy (%)	Precision (%)	Recall (%)	Accuracy (%)	Precision (%)	Recall (%)
5	92.35	88.57	91.67	85.29	81.87	80.19	80.35	83.91	83.33
10	90.29	90.15	91.82	92.76	96.54	81.12	82.47	86.26	77.78
15	96.11	90.76	91.82	93.55	95.55	90.24	88.75	86.11	85.85
20	98.42	95.71	100.00	97.38	96.34	91.63	94.28	95.13	94.29
25	94.75	91.76	91.76	94.34	92.28	80.05	90.83	94.28	90.60
30	92.28	90.91	83.33	89.75	89.86	88.33	89.57	89.68	88.38
35	92.85	93.33	100.00	92.85	87.35	83.13	89.57	91.75	79.19
40	90.31	85.71	87.42	88.22	82.24	86.37	85.23	81.74	87.22

By comparing the experimental results, among the three models used, SVM is more stable than AlexNet and MCNN, and the accuracy of test set and verification set is higher. The reason for the limited classification performance of AlexNet and MCNN may be the lack of data. Both AlexNet and MCNN require larger data sets to have a better generalization. In contrast, SVM requires only a small amount of data to have a good performance. Therefore, SVM is more suitable for classifying raisins than AlexNet and MCNN.

This experiment achieved better results than the results of Khojastehnazhandd et al. and Navab-Karimia et al. The possible reason is that the image processing reflects only the surface characteristics of raisins and does not allow the analysis of the internal structure of raisins. Infrared spectroscopy reflects information about chemical bonds or functional groups in the molecules of a substance, and therefore better shows the differences between different types of raisins.

## Conclusion

This study verified the feasibility of near-infrared spectroscopy combined with pattern recognition algorithms for adulterated raisins. We first analyzed the near-infrared spectroscopy images of the three kinds of raisins, and there were differences in the material content of the skins of different kinds of raisins. Then, we used SVM, MCNN, and the improved AlexNet model to classify raisins and got 100% accuracy. The experimental results show that though MCNN and AlexNet achieved good prediction results, SVM had a better classification effect on the skin of raisins. This experiment overcomes the limitations of the raisin image classification method and provides a simple, accurate, and fast method for the identification of raisin varieties. This method can also be applied to the detection of other granular foods. In addition, this experiment compared the classification capabilities of traditional machine learning algorithm and deep learning algorithms on small data sets and provided a certain idea for choosing a classification model.
